# 

**Published:** 1865-11

**Authors:** 


### CONTENTS.

### ORIGINAL CONTRIBUTIONS.

ART. XXXVII. Case of Schirrus. By R. S. Lewis, M.D., Dubuque, Iowa, ---- 641

ART. XXXVIII. Case of Poisoning with Arsenious Acid. By R. C. Hamill, M.D. Reported to the Chicago Medical Society. Published by request of the Society, ---- 643

### FOREIGN CORRESPONDENCE.

Brief Notes on the Cholera at Constantinople, 1865, ---- 645

Letter from Paris. By J., ---- 653

### PROCEEDINGS OF SOCIETIES.

Proceedings of Fox River Valley Association, ---- 658

### SELECTIONS.

The Food and the Teeth.—Observations on the Inorganic Constituents of the Food of Children, as connected with the Decay of the Teeth, and the Physical Constitution of Women in America. By the late James Paul, M.D., of Trenton, N.J., ---- 661

Report on the Use of Pressure in the Treatment of Gonorrhœal and Purulent Ophthalmia. By Jos. S. Hildreth, U.S.V., Desmarres (U. S. A.) Eye and Ear Hospital, Chicago, ---- 673

On Diphtheria. By Edward Headlam Greenhow, M.D., F.R.S.C.P., Assistant Physician to the Middlesex Hospital, &c., ---- 681

Medical Societies in Canada, ---- 688

On the Antagonism of Atropia and Morphia, founded upon Observations and Experiments made at the U.S.A. Hospital for Injuries and Diseases of the Nervous System. By S. Weir Mitchell, M.D., Wm. W. Keen, M.D., and Geo. R. Morehouse, M.D., ---- 689

### BOOK NOTICES.

The Practice of Medicine and Surgery Applied to the Diseases and Accidents incident to Women. By Wm. H. Byford, A.M., M.D., ---- 692

The Student's Book of Cutaneous Medicine and Diseases of the Skin. By Erasmus Wilson, F.R.S., ---- 693

Lectures on the Diseases of the Stomach, with an Introduction on its Anatomy and Physiology. By Williams Brinton, M.D., F.R.S., ---- 693

The Use of the Laryngoscope in Diseases of the Throat, &c. By Morrell Mackenzie, M.D., London, M.R.C.P., ---- 694

Researches of the Medical Properties and Applications of Protoxide of Nitrogen, or Laughing Gas. By Geo. J. Ziegler, M.D., ---- 694

Lectures on Inflammation, before the College of Physicians of Philadelphia. By John H. Packard, M.D., &c., ---- 694

Lectures on Fever, &c. By A. P. Merrill, M.D., &c., ---- 694

Physician's Visiting List, Diary, and Book of Engagements, for 1866, ---- 694

### EDITORIAL.

Duodeno-Hepatitis, or Acute Jaundice. By N. S. Davis, M.D., Prof. Clin. Medicine, &c., Chicago. The Substance of a Clinical Lecture in the Mercy Hospital, Oct. 23, 1865, ---- 694

Practical Pharmacy. By S. W. Gillespie, ---- 701

